# Cross-validation of the Student Perceptions of Team-Based Learning Scale in the United States

**DOI:** 10.3352/jeehp.2017.14.15

**Published:** 2017-06-29

**Authors:** Donald H. Lein, John D. Lowman, Christopher A. Eidson, Hon K. Yuen

**Affiliations:** 1 Department of Physical Therapy, School of Health Professions, The University of Alabama at Birmingham, Birmingham, Ala., USA; 2 Department of Occupational Therapy, School of Health Professions, The University of Alabama at Birmingham, Birmingham, Ala., USA; Hallym University, Korea

**Keywords:** Physical therapists, Perception, Educational measurement, Principal component analysis, Statistical factor analysis

## Abstract

**Purpose:**

The purpose of this study was to cross-validate the factor structure of the previously developed Student Perceptions of Team-Based Learning (TBL) Scale among students in an entry-level doctor of physical therapy (DPT) program in the United States.

**Methods:**

Toward the end of the semester in 2 patient/client management courses taught using TBL, 115 DPT students completed the Student Perceptions of TBL Scale, with a response rate of 87%. Principal component analysis (PCA) and confirmatory factor analysis (CFA) were conducted to replicate and confirm the underlying factor structure of the scale.

**Results:**

Based on the PCA for the validation sample, the original 2-factor structure (preference for TBL and preference for teamwork) of the Student Perceptions of TBL Scale was replicated. The overall goodness-of-fit indices from the CFA suggested that the original 2-factor structure for the 15 items of the scale demonstrated a good model fit (comparative fit index, 0.95; non-normed fit index/Tucker-Lewis index, 0.93; root mean square error of approximation, 0.06; and standardized root mean square residual, 0.07). The 2 factors demonstrated high internal consistency (alpha= 0.83 and 0.88, respectively). DPT students taught using TBL viewed the factor of preference for teamwork more favorably than preference for TBL.

**Conclusion:**

Our findings provide evidence supporting the replicability of the internal structure of the Student Perceptions of TBL Scale when assessing perceptions of TBL among DPT students in patient/client management courses.

## Introduction

Team-based learning (TBL) is an active-learning instructional strategy that was designed to promote individual and group accountability, collaborative learning, and acquisition of higher-order cognitive skills through application exercises [1]. Studies in medical and health professions education have shown that students’ knowledge scores improved after the implementation of TBL [2-4], and students demonstrated favorable perceptions and satisfaction with TBL [5]. Several measures have been developed to evaluate different aspects of students’ perceptions of TBL, including constructs related to accountability, preference, satisfaction, and team performance [6-9]. Of these measures, the Student Perceptions of TBL Scale developed by Vasan et al. [9] has been widely recognized [10], and, according to Scopus Citation Overview (https://www.scopus.com), has superseded the number of citations of similar TBL perception measures.

The student perceptions of TBL scale was originally developed from a 20-item questionnaire (related to perceptions of TBL and teamwork) completed by 2 cohorts of first-year medical students (N= 317) after they were taught using TBL in a gross anatomy course [9]. The content development of the 20 items was based on student feedback from 10 focus groups about their experience with TBL in the gross anatomy course. A principal component analysis (PCA) with varimax rotation was conducted to determine how these 20 items grouped together (i.e., factor structure). Two factors emerged; one contained 8 items that represented preference for TBL, with a Cronbach alpha coefficient of 0.91, and the other contained 7 items that represented preference for teamwork, with an alpha coefficient of 0.88. No confirmatory factor analysis (CFA) was conducted to support the internal structure of the 2 subscales. Evidence on relationships to other variables was confined to the relationship between 1) the estimation of anticipated course grades from students prior to the final exam and 2) scores of the 2 subscales [9].

It has been strongly advocated that the replication of evidence regarding the validity of the internal structure is essential in the process of developing and validating a psychometric instrument [11-13]. Evidence pertaining to the replication of internal structure requires that factor solutions within a particular data set be observed within another similar data set [12]. To replicate and confirm the underlying factor structure of the Student Perceptions of TBL Scale, it is necessary to evaluate this measure with a different sample in terms of student population, course content, and setting. The purpose of exploratory factor analysis (EFA) in replication is to provide a benchmark for the confirmation of factor structure of the existing instrument. CFA is used to evaluate the fit of the factor structure of the existing instrument when applied to a new set of data collected in another sample or context. Knafl and Grey [11] recommended supplementing a CFA with an EFA applied to the same data for comparison when conducting cross-validation.

Given the lack of studies investigating the replicability and generalizability of the factor structure of the Student Perceptions of TBL Scale in other relevant student populations, the purpose of this study was to cross-validate this scale in a group of entry-level doctor of physical therapy (DPT) students who took patient/client management courses taught using TBL. It was hypothesized that the original 2-factor structure of the Student Perceptions of TBL Scale would fit the observed data well.

## Methods

### Study design

A cross-sectional survey study was used to examine the replicability of evidence regarding the internal structure of the student perceptions of TBL scale.

### Participants

Entry-level DPT students, who were enrolled in either one of two 3-credit patient/client management courses (basic skills and cardiopulmonary) over 2 academic years (2012 and 2013), participated in this study. Of the 132 students in these courses, 115 completed the questionnaire (87% response rate). There were 85 first-year students from the basic skills course (43 first year students in 2012 and 42 in 2013) and 30 second-year students from the cardiopulmonary course (2012). All participants experienced TBL for the first time in their DPT curriculum.

### Ethical approval

The institutional review board of University of Alabama at Birmingham (Birmingham, Alabama, USA) approved the study protocol (X120206009).

### Procedure

The Student Perceptions of TBL Scale was administered in the classroom using a paper and pencil format toward the end of the semester in each course in which TBL was used throughout the semester.

### Measure

The Student Perceptions of TBL Scale is a 15-item instrument with 2 subscales: preference for TBL (8 items) and preference for teamwork (7 items). Each item was rated on a 5-point Likert-type scale: strongly disagree, −2; disagree, −1; neutral, 0; agree, 1; and strongly agree, 2, with a higher positive score indicating a more favorable perception toward TBL and teamwork [9].

### Data analysis

Based on the suggestions from Knafl and Grey [11], PCA and CFA were conducted to cross-validate the underlying factor structure of the Student Perceptions of TBL Scale. The Cronbach alpha was used to estimate the internal consistency reliability of the factors. A PCA factor-extraction method was conducted to replicate the dimensionality (i.e., number of components) of the 15 items of the Student Perceptions of TBL Scale. The use of the PCA to determine the factor structure was consistent with the selected extraction method used by Vasan et al. [9]. Prior to conducting the PCA, the KaiserMeyer-Olkin test (KMO; a measure of sampling adequacy or shared variance in the items) and the Bartlett sphericity test were conducted to check whether the sample met the criteria for factor analysis. The results showed that sampling adequacy was good, with a KMO value of 0.88 (above the commonly recommended value of 0.6), and the Bartlett test of sphericity was significant (P< 0.001), suggesting that the sample was suitable for factor analysis [14].

To determine the number of factors retained on the PCA, we used a scree plot and Horn parallel analysis. In Horn parallel analysis, eigenvalues from the PCA solution are compared to eigenvalues from a randomly generated data matrix of the sample (i.e., 15 items×115 respondents in this study). The eigenvalues larger than those of the randomly generated data are used to determine the number of factors retained [14]. An examination of the scree plot revealed a clear break after the second component; therefore, retaining 2 factors was appropriate for the data. The 2-factor solution, consistent with the findings of Vasan et al. [9], was verified using Horn parallel analysis, as there were only 2 components with eigenvalues exceeding those of the randomly generated data. As factors emerging from the data were expected to be correlated, a promax oblique rotation method was used to achieve a simpler structure for interpretation. An item was considered to be important in explaining the amount of variance of a factor if its factor loading was ≥0.4 (as an absolute value) [11]. PCA was conducted using IBM SPSS for Windows ver. 23.0 (IBM Corp., Armonk, NY, USA).

CFA was performed to test the suitability of the 2-factor model previously identified by Vasan et al. [9]. The sample variance-covariance matrix of the 15 items was evaluated using a maximum-likelihood minimization function to validate the 2-factor model. Model fit statistics, factor loadings, and modification indices were inspected to determine whether the 2-factor model provided an acceptable fit to the observed data. The goodness-of-fit of the CFA model was evaluated using the comparative fit index (CFI), non-normed fit index (NNFI)/Tucker-Lewis index (TLI), root mean square error of approximation (RMSEA), and standardized root mean square residual (SRMR). In general, values of CFI ≥ 0.95, NNFI/TLI ≥ 0.95, RMSEA ≤ 0.07, and SRMR ≤ 0.08 are indicative of good fit with acceptable CFA models [14]. CFA was conducted using the LISREL ver. 9.2. software program (Scientific Software International Inc., Lincolnwood, IL, USA).

## Results

Among the 115 student participants who completed the questionnaire, 82 (71%) were female, and 107 (93%) were Caucasian. The mean and standard deviation of the participants’ age was 24±3 years, ranging from 21 to 40 years. Raw data were available from Supplement 1.

After the promax rotation, 8 items representing one component (preference for TBL) and another 7 items representing the other component (preference for teamwork) with factor loadings ≥ 0.4 on each emerged; no items demonstrated cross-loading (i.e., having factor loadings of ≥ 0.4 on both components) [14]. Together, these 2 factors of the Student Perceptions of TBL Scale accounted for 55% of the total variance; the first factor explained 43% of the variance (eigenvalue= 6.5) and the second explained 12% of the variance (eigenvalue= 1.8). The factor-loading matrix and commonalities, along with the percentage of variance, eigenvalues, and Cronbach alpha coefficients for this final solution are presented in Table 1.

**Fig. 1.**
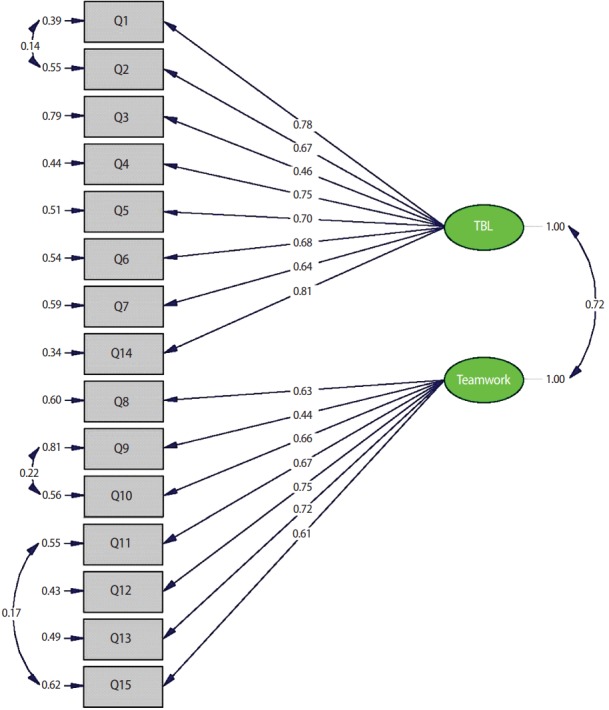
Confirmatory factor model for the Student Perceptions of TBL Scale. TBL, team-based learning. Chi-square = 125.79; df = 86; P-value = 0.00335; root mean square error of approximation = 0.063.

Regarding the CFA, all items loaded significantly onto their respective factors, with loadings ranging from 0.46 to 0.81 on the preference for TBL subscale and 0.44 to 0.76 on the preference for teamwork subscale. All freely estimated unstandardized parameters were statistically significant, with P-values < 0.001. Factor loading estimates indicated that the 15 items were moderately related to their proposed factors (R^2^ : 0.19–0.66), suggesting that the Student Perceptions of TBL Scale was a reasonably reliable indicator of the constructs of preference for TBL and preference for teamwork. The chisquare value for the overall model was significant (χ^2^ (89)= 154.43, P< 0.001), suggesting a lack of fit between the data and the hypothesized model. Modification indices suggested that allowing correlation of the 3 error covariances (i.e., freeing the covariance between 3 pairs of error terms: items 1 and 2, items 9 and 10, and items 11 and 15) would improve the model fit. A model including these 3 specific correlations resulted in a subsequent model having better fit to the constrained model (χ^2^ (86)= 125.79, P= 0.003), with CFI= 0.95, NNFI/TLI= 0.93, RMSEA= 0.06, and SRMR= 0.07. The χ^2^ difference test indicated that the constrained model fit the data significantly better than the unconstrained model, (Δχ^2^ (3)=28.64, P<0.001); therefore, the constrained model was adopted as the final model (Fig. 1).

The grand item mean and standard deviation of the 2 factors (8 items for preference for TBL and 7 items for preference for teamwork) were 0.67± 0.64 and 1.30± 0.54, respectively. The paired-samples t-test indicated that DPT students taught using TBL viewed preference for teamwork significantly more favorably than preference for TBL (t(114)= 12.56, P< 0.001). The internal consistency reliability was 0.88 (95% confidence interval [CI], 0.84 to 0.91) for preference for TBL as estimated by the Cronbach alpha, and 0.83 (95% CI, 0.77 to 0.87) for preference for teamwork, which are both considered to be good. There was no noticeable increase in the alpha coefficient (< 0.5%) for the 2 subscales when an item was eliminated from its respective subscale. The corrected item-to-total correlations between scores of an individual item and the summation score of the remaining items in each of the 2 factors were all above 0.4 (moderate correlation). The internal consistency reliability of the whole scale was 0.90 (95% CI, 0.87 to 0.93). Estimates from the 2-factor solution indicated a strong association between the factors of preference for TBL and preference for teamwork (r=0.72, P< 0.001), supporting the proposition that the 2 subscales are related.

## Discussion

Based on the PCA, the 15 items on the Student Perceptions of TBL Scale clustered into 2 components, which replicated the 2-factor structure identified by Vasan et al. [9]. The overall goodness-of-fit indices from the CFA suggested that the original 2-factor structure for the 15 items of the Student Perceptions of TBL Scale demonstrated good model fit, thus providing evidence to support the internal structure of this scale that has been applied to assess perceptions of TBL among DPT students in patient/client management courses. The 2 factors in this study demonstrated high internal consistency; and the alpha coefficients were comparable to those reported in the original study of Vasan et al. [9] (0.88 in the present study versus 0.91 in the study of Vasan et al. [9] on preference for TBL, and 0.83 in the present study versus 0.88 in the study of Vasan et al. [9] on preference for teamwork). In addition, our results are consistent with those of previous studies of medical and health professions students receiving TBL instructional methods, in that students viewed preference for teamwork more favorably than preference for the TBL process [9,15,16].

### Limitations and future directions

Our study has several limitations. The relatively small sample size is one of the biggest limitations. Even though our sample size was adequate for stable and precise estimates of population loadings, a larger sample size (e.g., ≥ 300) may be needed to obtain a good recovery of population factors [17]. Second, our sampling method was non-probabilistic, and therefore may or may not reflect the characteristics of the DPT student population in the United States. Future studies with larger samples and probabilistic sampling procedures are needed to reduce biases in estimating the scores of this instrument. Moreover, future studies may examine the factor structure of this instrument in college student samples with different ages, racial/ethnic groups, and/or cultural backgrounds. Multi-group analyses may provide valuable information on measurement invariance of the construct of this instrument across ages, races/ethnicities, or cultures. While the initial evidence of reliability and internal structure of this instrument is encouraging, other sources of evidence regarding relationships to other variables are needed to show that the scale measures the constructs of student preferences (for TBL and teamwork) in an adequate way. Such variables and outcomes may include students’ actual academic grades and scores (attitudes/perceptions, skills, and/or behaviours) of tools measuring teamwork in the classroom, simulations, and/or clinical settings [10].

### Implications and conclusions

This study validated an instrument that provides educators with a way to quantitatively assess student preferences for TBL and team interactions. Consequently, this tool will be useful for evaluating the instructional method of TBL and different components within it, including group processes among students in health professions academic courses.

Despite differences in characteristics (professions, course, and institution) across the samples, our findings confirmed the replicability of the 2-factor structure of the Student Perceptions of TBL Scale with a good model fit in a different sample (DPT students in patient/client management courses) from previous research. The factor structure might be applicable to other courses and different student samples in other academic settings, but measurement invariance of the construct needs to be confirmed. In conclusion, the findings of the present cross-validation study provided robust evidence strengthening the psychometric properties of the Student Perceptions of TBL Scale.

## Figures and Tables

**Table 1.** Factor loadings and communalities based on principal component analysis with promax rotation for the 15 items on the Student Perceptions of TBL Scale (N = 115)

Item no.	Item description	TBL	Teamwork	Communality	Mean ± standard deviation
1	TBL helped me increase my understanding of the course material.	0.85		0.69	0.51±0.88
2	Learning issues helped me to focus on core information.	0.84		0.62	0.56±0.76
3	Individual readiness assurance tests were useful learning activities.	0.73		0.41	0.66±0.89
4	Discussions of the TBL learning issues were useful learning activities.	0.70		0.60	0.93±0.80
5	I learned useful additional information during the TBL sessions.	0.78		0.58	0.62±0.91
6	TBL helped me prepare for course examinations.	0.68		0.49	0.39±1.03
7	The Group Readiness Assurance Test (group) discussions allowed me to correct my mistakes and improve understanding of the concepts.	0.40		0.47	1.20±0.80
14	The TBL format was helpful in developing my information synthesizing skills.	0.69		0.65	0.51±0.91
8	I have a positive attitude about working with my peers.		0.85	0.60	1.47±0.63
9	The ability to collaborate with my peers is necessary if I am to be successful as a student.		0.41	0.25	1.37±0.85
10	Solving problems in a group is an effective way to practice what I have learned.		0.50	0.52	1.17±0.76
11	My team worked well together.		0.88	0.65	1.57±0.68
12	I contributed meaningfully to the TBL discussions.		0.64	0.56	1.26±0.65
13	Most students were attentive during TBL sessions.		0.58	0.52	0.75±1.03
15	There was mutual respect for other teammates' viewpoints during TBL.		0.87	0.62	1.50±0.69
	Eigenvalues	6.45	1.78		
	% of variance	43	12		
	Cronbach alpha	0.88	0.83		

TBL, team-based learning.

## Supplementary materials

Supplement 1. Data file is available from https://dataverse.harvard.edu/dataset.xhtml?persistentId=doi%3A10.7910%2FDVN%2FCGLH88

Supplement 2. Audio recording of the abstract.
